# Promotion of Proangiogenic Secretome from Mesenchymal Stromal Cells via Hierarchically Structured Biodegradable Microcarriers

**DOI:** 10.1002/adbi.202000062

**Published:** 2020-06-08

**Authors:** Chara Simitzi, Eseelle Hendow, Zhuangnan Li, Richard M. Day

**Affiliations:** ^1^ Applied Biomedical Engineering Group Centre for Precision Healthcare UCL Division of Medicine University College London London WC1E 6JF UK; ^2^ Department of Chemistry University College London London WC1H 0AJ UK

**Keywords:** angiogenesis, mesenchymal stromal cells secretome, PLGA microcarriers

## Abstract

Adipose‐derived mesenchymal stromal cells (AdMSC) release numerous soluble factors capable of stimulating angiogenesis. Improved methods for delivering these cells to maximize their potency are now sought that ideally they retain viable cells in the target tissue while promoting the secretion of angiogenic factors. Substrate surface topography is a parameter that can be used to manipulate the behavior of AdMSC but challenges exist with translating this parameter into materials compatible with minimally invasive delivery into tissues for in situ delivery of the angiogenic secretome. The current study investigates three compositions of hierarchically structured, porous biodegradable microcarriers for the culture of AdMSC and the influence of their surface topographies on the angiogenic secretome. All three compositions perform well as cell microcarriers in xeno‐free conditions. The attached AdMSC retain their capacity for subsequent trilineage differentiation. The secretome of AdMSC attached to the microcarriers consists of multiple proangiogenic factors, including significantly elevated levels of vascular endothelial growth factor, which stimulates angiogenesis in vitro. The unique properties of hierarchically structured, porous biodegradable microcarriers investigated in this study offer a radically transformative approach for achieving targeted in vivo delivery of AdMSC and enhancing the potency of their proangiogenic activity to induce neovascularization in ischemic tissue.

## Graphical abstract

This study investigates highly textured microspheres designed for use as implantable cell microcarriers that are shown to stimulate the secretion of proangiogenic factors from attached mesenchymal stromal cells. These findings suggest that the technology can provide a feasible and transformative new approach for treating cardiovascular disease through the targeted in situ delivery of factors that will promote new blood vessel growth.

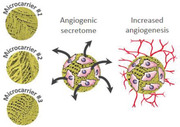

## 1 Introduction

Mesenchymal stromal cells (MSC) secrete a plurality of molecules, collectively called the MSC secretome, which include a heterogeneous pool of soluble proteins, free nucleic acids, lipids and extracellular vesicles that have effects on different cell populations (reviewed in ref. [1]). The MSC secretome is reported to include numerous growth factors, with antiapoptotic,^[^ ^2^ ^]^ mitogenic^[^ ^3^ ^]^ proangiogenic^[^ ^2^, ^4^, ^5^ ^]^ and anti‐inflammatory^[^ ^6^ ^]^ action. In relation to cardiovascular disease, the MSC secretome has been reported to influence angiogenesis in vitro^[^ ^4^, ^5^, ^7^ ^]^ and in vivo,^[^ ^8^ ^]^ facilitating revascularization, immune modulation, wound healing and tissue repair.^[^ ^3^ ^]^ Due to the potent trophic and paracrine immunomodulatory activity of MSC, there is an increasing interest in exploiting the MSC secretome for new therapeutic strategies (reviewed in ref. [1, 9]).

The paracrine activity of MSC is affected by the local cellular microenvironment, which comprises a plethora of biochemical, mechanical and physical cues (reviewed in ref. [10]). Soluble factors, such as transforming growth factor (TGF) b^[^ ^11^ ^]^ or lipopolysaccharide^[^ ^12^ ^]^ can be sensed by MSC and subsequently alter their paracrine activities. Furthermore, physiological manipulation, such as exposure to hypoxic conditions, has been shown to increase secretion of certain growth factors, such as vascular endothelial growth factor (VEGF), fibroblast growth factor 2 (FGF‐2), hepatocyte growth factor (HGF) and anti‐inflammatory molecules (reviewed in ref. [13]). The mode of cell culture may also influence the MSC secretome. Specifically, 3D cell culture in the form of multicellular spheroids results in elevated secretion profiles of proangiogenic, anti‐inflammatory and/or antiapoptotic factors compared with the secretome derived from conventional monolayer culture.^[^ ^14^ ^]^ Furthermore, mechanical stimulation (such as bioreactor‐mediated mechanical loading) of MSC results in a significant enhancement of their proangiogenic paracrine activity compared with unstimulated MSC.^[^ ^15^ ^]^ Increased understanding of the physicochemical cues that influence the MSC secretome is a prerequisite to harnessing the paracrine activity of these cells for therapeutic applications in cardiovascular disease,^[^ ^16^ ^]^ cancer,^[^ ^9^ ^]^ neurodegenerative diseases^[^ ^17^ ^]^ and regenerative medicine.^[^ ^18^ ^]^

Biomedical engineering has made possible the development of novel cell culture substrates that provide instructive cues at a cellular and subcellular level and has highlighted potential new opportunities for harnessing the therapeutic effect of MSC. Examples include cues on 2D substrates, such as topographical, geometrical and mechanical features that affect MSC growth and lineage specification,^[^ ^19^, ^20^, ^21^, ^22^ ^]^ through to the development of elaborate cell culture platforms with imprinted physical cues used to study the mechanisms underpinning environmental sensing by MSC and control of their behavior.^[^ ^21^, ^23^, ^24^ ^]^ More recently, investigators have started to study the MSC secretome in response to such cues.^[^ ^25^, ^26^, ^27^, ^28^, ^29^, ^30^ ^]^ However, to date, there has been a paucity of new technology compatible with easily delivering these cues in vivo and that is compatible with existing, scalable platforms used in bioprocessing to ease future translation.

Polymeric microcarriers are widely used as cell substrates during the expansion of MSC in scale‐up bioreactor systems.^[^ ^31^ ^]^ Cell attachment to microcarriers can be enhanced by inclusion of instructive cues, such as physical features (e.g., porosity) and biochemical properties (e.g., surface chemistry charge or protein coating), as reviewed elsewhere).^[^ ^32^ ^]^ However, existing commercial microcarriers are not suitable for clinical translation. Therefore, significant opportunities exist for the creation of new, smart biomaterial‐based microcarrier substrates that combine cell instructive cues in a format that can be integrated with the extensive technology of that already exists for culturing microcarriers and are biocompatible for in vivo implantation. Such technology would deliver a transformative approach for targeted 3D substrate‐induced manipulation of the MSC secretome in situ.

The current study reports for the first time on biodegradable microcarriers with unique hierarchical porosity designed to manipulate MSC toward a proangiogenic secretome. Three compositions of microcarriers with distinct surface topographies were fabricated using thermally induced phase separation (TIPS), a process that enables infinite adjustment to the hierarchical surface topography.^[^ ^33^ ^]^ All three TIPS microcarrier types performed well as cell microcarriers, while cell attachment of AdMSC on solid microparticles made of the same polymer was very limited. Data from the study demonstrate that attachment and culture of human AdMSC on the surface of TIPS microcarriers in xeno‐free conditions results in an enhanced proangiogenic secretome. This finding may provide a new approach for enhancing the paracrine activity of MSC for use in revascularizing ischemic tissue.

## 2 Results

### 2.1 Fabrication and Characterization of Poly‐d,l‐lactide‐*co*‐glycolide TIPS Microcarriers

**Figure 1:**
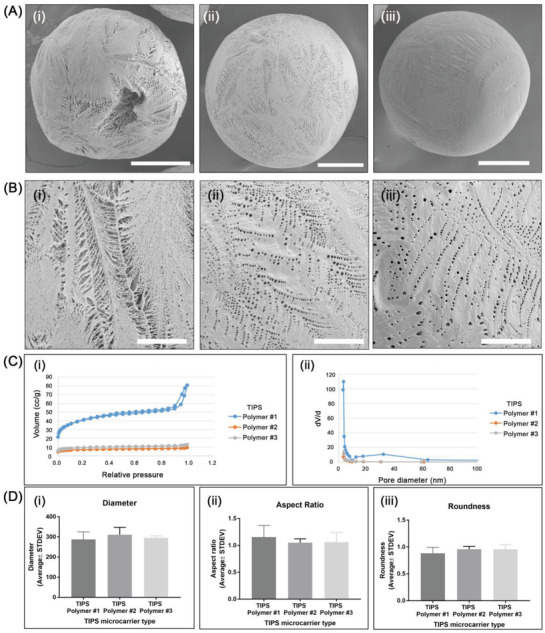
Porous PLGA microcarriers fabricated by thermally induced phase separation (TIPS). A) SEM images of TIPS microcarriers of polymers 1 i), 2 ii), and 3 iii) at low and B) high magnification (scale bar in A is 100 µm; in B is 25 µm). C) Evaluation of porosity of TIPS microcarriers measured by nitrogen adsorption porosimetry: i) nitrogen adsorption/desorption isotherms (for polymers 2 and 3 the hysteresis loop between the adsorption and desorption curves is very small, so that the two curves are shown as one) and ii) pore size distributions. D) Quantitative evaluation of morphological characteristics of the PLGA TIPS microcarriers: i) diameter, ii) aspect ratio, and iii) roundness.

The porous biodegradable TIPS microcarriers were fabricated using thermally induced phase separation. The manufacturing process has previously been shown to be simple, robust, and highly tunable, enabling control of size, surface topography, and degradation rate.^[^ ^33^, ^34^ ^]^ In the current study, three different compositions of Poly‐d,l‐lactide‐*co*‐glycolide (PLGA) were used to create three types of TIPS microcarriers (denoted as polymer 1, polymer 2, and polymer 3). All three polymers produced microcarriers that exhibited hierarchical pore structure characteristic of the TIPS process,^[^ ^33^ ^]^ but each exhibited different features of surface topography and porosity (**Figure** **1**A). The differences in surface porosity were more apparent at higher magnification scanning electron microscopy (SEM) (Figure 1B). TIPS microcarriers composed of polymer 1 were highly porous, and more porous compared with the TIPS microcarriers of polymers 2 and 3 (Figure S1i, Supporting Information). Pores of the TIPS microcarrier of polymer 1 had both circular and longitudinal shape, as shown from the high aspect ratio and low circularity values, while the pores of the TIPS microcarriers of polymers 2 and 3 were mainly circular (Figure S1ii,iii, Supporting Information). TIPS microcarriers of polymer 3 exhibited the smoothest surface. Nitrogen adsorption porosimetry revealed that microcarriers composed of polymer type 1 were highly porous, having the largest Brunauer–Emmett–Teller (BET) surface area of 145.2 m^2^ g^−1^ (Figure 1Ci,ii). In comparison, the other two types of TIPS microcarrier were relatively less porous (BET surface area of 27.6 and 33.1 m^2^ g^−1^ for microcarriers composed of polymer type 2 and type 3, respectively).

The mean diameter of all three types of TIPS microcarriers was ≈300 µm (Figure 1D). TIPS microcarriers composed of polymer 1 were less spherical and had a slightly (nonsignificant) higher aspect ratio compared with the microcarriers composed of polymers 2 and 3 (Figure 1D). Prewetting of TIPS microcarriers prior to cell attachment resulted in a reduction in the mean diameter without influencing their shape. The most porous composition of microcarrier (polymer 1) had the greatest decrease in diameter (≈45.0%), followed by polymer 2 (≈28.2%), and polymer 3 (≈16.8%).

Solid PLGA microcarriers were included in the study as nontextured controls. All three compositions of polymer were compatible with the emulsion solvent evaporation technique for producing solid microcarriers; however, polymers 2 and 3 were most suited for achieving solid microcarriers similar to the size range of TIPS microcarriers (Figure S2, Supporting Information).

For the angiogenic secretome screening experiment, solid polystyrene (PS) microcarriers (PS300K, Phosphorex) have been used as additional control. The size range of these microcarriers was 250–355 µm (mean diameter 306.2 ±2 9.6 µm).

### 2.2 Evaluation of AdMSC Culture on TIPS Microcarriers

**Figure 2:**
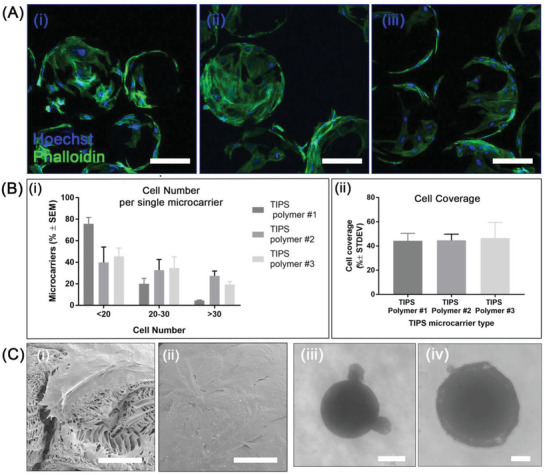
Cell adhesion of AdMSC on TIPS microcarriers. A) Confocal microscopy images of phalloidin (green) and Hoechst (blue) stained AdMSC attached to the surface of TIPS microcarriers composed of polymer 1 i), polymer 2 ii), and polymer 3 iii) microcarriers after 6 h incubation. Scale bar: 100 µm. B) Quantitative evaluation of cell adhesion on TIPS microcarriers after 6 h. i) relative quantity of TIPS microcarriers with cells attached within a specific range (data from three experiments; *n* = 70–90 TIPS microcarriers per type and experiment). ii) Surface coverage of microcarriers by cells per single TIPS microcarrier. Coverage is expressed as mean % ± standard deviation (STDEV) (≈120 TIPS microcarriers measured per polymer type). C) SEM images of AdMSC adhered to the surface of a TIPS microcarrier composed of polymer 1 for 24 h i,ii) (scale bar in Ci is 20 µm; scale bar in Cii is 50 µm); iii,iv) brightfield microscopy images of AdMSC attached to a single microcarrier in the hanging drop culture at 48 h postseeding: iii) a solid microcarrier and iv) TIPS microcarrier composed of polymer 3 (scale bar in Ciii and Civ is 100 µm).

To investigate the seeding efficiency of AdMSC on the different types of TIPS microcarrier, 0.5 × 10^6^ AdMSC were cocultured with ≈17 500 TIPS microcarriers under semidynamic incubation conditions. Within the first 6 h, cells attached and began spreading on the surface of all compositions of TIPS microcarriers (**Figure** **2**A). Cell coverage of the microcarrier surface was ≈45% of the surface area for all three types of TIPS microcarriers (Figure 2Bii). At 6 h postseeding a higher proportion of TIPS microcarriers composed of polymer 1 had less than ≈20 cells per microcarrier compared with polymers 2 and 3. The proportion of microcarriers with 20–30 or >30 cells per microcarrier was similar for polymers 2 and 3, but greater than polymer 1 (Figure 2Bi). SEM showed complete coverage of cells on the surface of all three types of TIPS microcarriers after 24 h culture under semidynamic conditions, with evidence of cell infiltration into the porous structure of the TIPS microcarriers (Figure 2Ci,ii).

Parallel experiments with the solid PLGA microcarriers showed very limited adhesion of the AdMSC. Cells cocultured with individual microcarriers in hanging drop plates revealed clusters of AdMSC at the surface of the solid microcarriers (Figure 2Ciii) whereas cells cocultured with TIPS microcarriers formed a homogenous cell monolayer over the surface of the microcarrier (Figure 2Civ).

### 2.3 Evaluation of Cell Growth of AdMSC on TIPS Microcarriers

**Figure 3:**
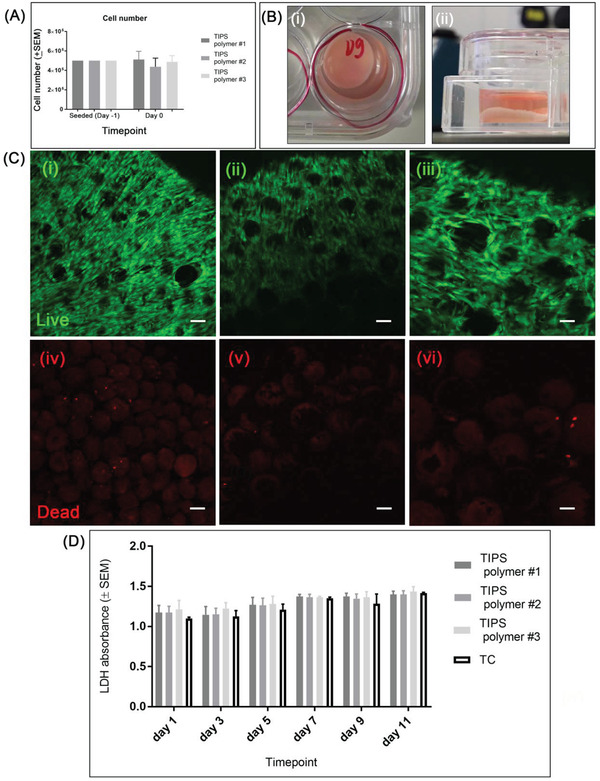
Cell growth of AdMSC on PLGA TIPS microcarriers for long‐term culture. A) Quantitative evaluation of cell attachment efficiency on the PLGA‐TIPS microcarriers after 24 h under semidynamic culture conditions; the results are expressed as mean cell number (±standard error of the mean, SEM; *n* = 3 experiments). B) 3D cellularized TIPS microcarrier construct viewed from above i) and side ii) at day 7. C) Confocal microscopy images of live (calcein: green) and dead (ethidium homodimer‐1: red) AdMSC after 11 days of culture on PLGA TIPS polymer 1 i,iv), polymer 2 ii,v), and polymer 3 iii,vi) microcarriers (scale bar in C is 100 µm). D) LDH absorbance expressed as mean relative absorbance (sample values are normalized by culture medium value) ± SEM (*n* = 3 experiments).

The cellularized microcarriers were cultured in vitro for up to 11 days under static conditions in 24‐well low bind plates, with the supernatants collected at 2 day intervals for subsequent analysis. At 24 h the majority of seeded AdMSC had attached to the surface of all three compositions of TIPS microcarriers (**Figure** **3**A). When confined within the 24‐well low bind plates under static incubation conditions the cellularized microcarriers fused together and formed a self‐supporting 3D disc by day 7 (Figure 3B). At day 11, cells were forming visible bridges between adjacent microcarriers. Live/Dead fluorescence staining of the 3D construct revealed predominantly viable cells throughout with very few dead cells (Figure 3C).

Measurement of lactate dehydrogenase (LDH) activity in the supernatants collected at different time points indicate the level of cell viability remained high throughout the study period and was comparable to the level measured for cells cultured on tissue culture plastic (Figure 3D). Comparing the values at day 1 and day 11, a nonsignificant increase in LDH absorbance occurred for the TIPS microcarrier of polymer 3, while a significant increase occurred for the TIPS microcarriers of polymers 1 and 2 (*p < 0.05). There were no statistically significant differences among the three types of TIPS microcarriers.

After 11 days in culture, MSC that had migrated from the microcarriers onto tissue culture plastic retained their lineage plasticity and could differentiate toward adipogenic, osteogenic and chondrogenic lineage (Figure S3, Supporting Information).

### 2.4 Angiogenic Secretome from Cellularized TIPS Microcarriers

**Figure 4:**
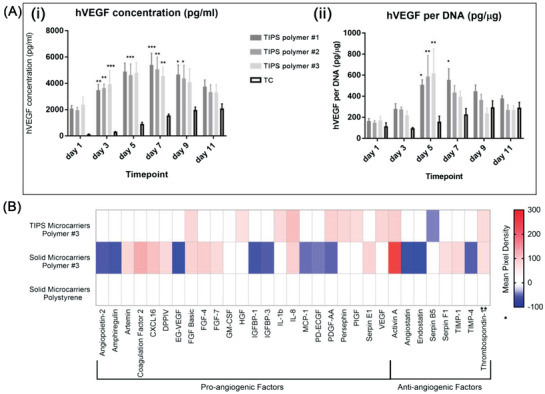
Evaluation of the angiogenic secretome from AdMSC cultured on TIPS microcarriers. A) Human VEGF in the secretome of AdMSC cultured on different compositions of TIPS microcarriers or tissue culture (TC) plastic. The results are presented as i) VEGF concentration (pg mL^−1^) and ii) adjusted for VEGF per DNA (pg µg^−1^). The level of significance was calculated using the one‐way ANOVA followed by Tukey test for multiple comparisons between the “TC” condition and the test samples (TIPS microcarriers) (*p < 0.05, **p < 0.01, and ***p < 0.001; the data represent three independent experiments). B) The relative amount of soluble angiogenesis‐related proteins in the pooled supernatants from cellularized TIPS microcarriers and solid microcarriers composed of polymer 3 and PS solid microcarriers using the human proteome profiler angiogenesis array. Heat maps for the analytes were generated by quantifying the mean spot pixel densities from two separate experiments in the array membrane using image software. The data were normalized against the values obtained for the control PS microcarriers.

The concentration of VEGF was higher in supernatants collected from each composition of cellularized TIPS microcarrier compared with the supernatants collected from cells cultured on tissue culture plastic at all timepoints (**Figure** **4**Ai). The concentration of VEGF measured in the supernatant indicated a nonmonotonic response, with the highest concentration detected at days 5–7. When each group was adjusted for the number of cells per condition (via the amount of DNA), the concentration of VEGF was significantly higher in supernatants collected from cellularized TIPS microcarriers at days 5 and 7 compared with cells cultured on tissue culture plastic (Figure 4Aii).

A semiquantitative assessment of the plurality of angiogenic factors secreted by AdMSC cultured on TIPS microcarriers was further investigated with microcarriers composed of polymer 3. The angiogenic proteome profiler array indicated the secreted levels of several other proangiogenic factors, including basic fibroblast growth factor (FGF),^[^ ^35^ ^]^ HGF,^[^ ^36^ ^]^ platelet derived growth factor (PDGF‐AA),^[^ ^37^ ^]^ placental growth factor‐1 (PIGF),^[^ ^38^ ^]^ IL‐1β^[^ ^39^ ^]^ and VEGF were increased in response to AdMSC culturing on the surface of TIPS microcarriers compared with AdMSC cultured on control microcarriers composed of solid polymer 3 or PS (Figure 4B).

**Figure 5:**
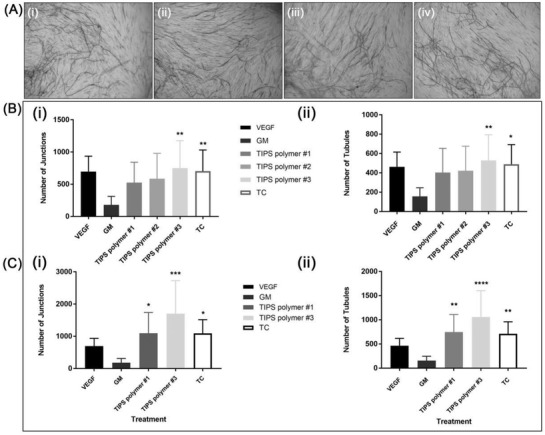
A) Light microscopy images of CD31 positive endothelial tubules following incubation in supernatant collected from AdMSC cultured on TIPS microcarriers composed of polymers 1 i), 2 ii), and 3 iii), and from AdMSC cultured on tissue culture (TC) plastic iv) for 14 days. B) Quantification of capillary‐like tubules following incubation in supernatants collected from cellularized TIPS microcarriers or cells cultured on tissue culture (TC) plastic. Conditioned medium from the AdMSC on TIPS microcarriers or the TC was collected at 2 day intervals from day 1 postseeding for a total of 10 days (corresponding to samples from days 1–11 in the VEGF ELISA presented in Figure 4). The conditioned medium was added to the V2a assay at 2 day intervals. i) Number of junctions and ii) number of tubules at day 14. C) Quantification of capillary‐like tubule formation in response to supernatants from cellularized TIPS microcarriers composed of polymers 1 and 3 or TC collected at day 3 only. The conditioned medium was added to the V2a assay at 2 day intervals. i) Number of junctions and ii) number of tubules at day 14. Data expressed as mean number (±standard deviation). Level of significance level calculated using the one‐way ANOVA followed by Tukey test for multiple comparisons between wells incubated in assay “growth medium” (GM) and test samples (*p* < 0.05; **, *p* < 0.01, *p* < 0.001, and ****p < 0.0001; the data represent three replicates per condition).

To evaluate whether the secretome from cellularized TIPS microcarriers was proangiogenic, the collected supernatants were further tested in vitro using an angiogenesis assay that enabled quantification of capillary‐like tubule formation. At day 14, tubule formation was visible in wells incubated with supernatants collected from all compositions of the cellularized TIPS microcarriers (**Figure** **5**A). For wells incubated with supernatant collected from microcarriers composed of polymer 3, the number of tubules and junctions was higher compared with control wells treated with 2 ng VEGF (Figure 5B). The supernatants collected from cells incubated on tissue culture plastic also produced a stimulatory effect.

To investigate the potency of supernatants collected from a single time point, a parallel angiogenesis assay was conducted using supernatants collected from TIPS microcarriers composed of polymers 1 and 3 at day 3. The supernatants collected from both cellularized microcarriers at the single time point resulted in the formation of a greater number of tubules and junctions (Figure 5C) compared with the supernatants collected from the same type of microcarriers at sequential time points (Figure 5B).

## 3 Discussion and Future Perspectives

### 3.1 Discussion

The paracrine action mediated MSC represents an attractive tool for a range of cell‐based therapies. While many clinical studies have endeavoured to utilize this effect, existing modes of application are limited to delivering the inherently anchorage dependent cell type in a suspended formulation, detached from a substratum. A radically different approach for harnessing the paracrine secretion of MSC would consist of delivering the cells in a more natural state, i.e., attached to an implantable substrate, which would also provide opportunities to refine the surface to include cues that enhance specific components of the secretome.

To explore this concept, the current study investigated whether highly porous, biodegradable PLGA TIPS microcarriers could be used for culture of AdMSC and manipulation of their secretome toward proangiogenic activity. A key feature of the microcarriers investigated in the current study is their potential for use as an implantable cell substrate. PLGA has an excellent clinical safety record and has been successfully used in a range of medical devices and drug delivery systems. TIPS microspheres have developed as a scaffold device for repair of perianal fistulas and are currently under clinical investigation.^[^ ^40^ ^]^ It is therefore feasible that PLGA TIPS microcarriers could be translated into clinical use for targeted delivery of AdMSC and to provide an effective stimulus for the secretion of proangiogenic growth factors. This would offer a transformative approach to harness the effect of the AdMSC secretome in a therapeutic setting.

Compared with other techniques for the fabrication of porous microspheres, the TIPS process is simple, robust and does not require the addition of porogen material. The size and morphology of TIPS microcarriers produced for the investigation were readily controlled by parameters related to the polymer solution (e.g., polymer MW and viscosity, concentration) and to the process (e.g., nozzle orifice, polymer flow rate, and distance between the nozzle and the bath). The method described in the current study enabled the fabrication of biodegradable microcarriers from three different compositions of PLGA that exhibited visibly different surface topographies. TIPS microcarriers of polymer 1 were highly porous compared with the microcarriers of polymers 2 and polymer 3. Microcarriers composed of polymer 1 underwent a greater reduction in size following the wetting process used to facilitate cell attachment compared with microcarriers composed of polymers 2 and 3. However, all three types of TIPS microcarriers were found to support equivalent cell adhesion and growth of AdMSC, with no significant difference observed between any of the microcarrier compositions.

After 1 day of culture, the surface of the TIPS microcarriers were fully covered by a monolayer of AdMSC. On the contrary, AdMSC failed to adhere uniformly to the surface of the solid PLGA microcarriers that exhibited a smooth surface. The importance of surface topography on cell attachment is well recognized for other cell culture platforms. For example, the impact of surface topography on cell adhesion to polymeric microspheres has been described elsewhere, with limited cell adhesion occurring with solid microspheres compared with the microspheres exhibiting a textured surface.^[^ ^41^, ^42^, ^43^, ^44^ ^]^ Similar phenomena account for the need to apply surface coatings to commercially available nontextured microcarriers to enhance cell attachment. The cell attachment and spreading behavior observed with TIPS microcarriers aligns with the notion that textured surfaces are conducive to supporting cell attachment and spreading.

The cell substratum plays a key role in controlling MSC differentiation. Besides biochemical soluble cues, there is increasing evidence that physical cues, including topographical features^[^ ^22^, ^45^ ^]^ and stiffness^[^ ^20^, ^46^ ^]^ also regulate lineage specification of MSC. On the contrary, the impact of the extracellular environment on the MSC secretome is an emerging field. Among the various cues that affect the paracrine activity of MSC, biochemical cues are better studied and understood. The effect of physical cues such as the topography of the cell substrate on the paracrine activity remains limited. Biomaterials that simulate physical cues experienced by MSC in their native environment might offer great potential of manipulating their secretome over conventional planar 2D cell culture substrates such as tissue culture plastic. Evidence in support of this concept includes preformed electrospun scaffolds composed of fibers in random, aligned and mesh‐like patterns that resulted in AdMSC producing significantly higher levels of anti‐inflammatory and proangiogenic factors compared with cells cultured on tissue culture microplates.^[^ ^28^ ^]^ Similarly, a comparison of flat substrates and 3D PS substrates resulted in differences in the crosstalk between MSC and endothelial cells or MSCs and human osteoblasts.^[^ ^47^ ^]^ Such studies suggest a biomaterial‐based approach is feasible for manipulating the paracrine activity of the MSC and, thus, increase opportunities for therapeutic applications. However, to be practicable for the broad range of uses where MSC are being investigated for their paracrine activity, rather than tissue replacement strategies, new approaches compatible with minimally invasive delivery are required. Implantable biodegradable microcarriers provide an ideal solution for this, since they provide a temporary cell substrate for delivery of cells in their anchored, natural state. Moreover, unlike other preformed scaffolds, the physical format of implantable microcarriers provide a formulation that can be delivered minimally invasively and conforms to the shape of the implant site.

In the current study, a trend toward greater proangiogenic secretome was observed when AdMSC were cultured on PLGA TIPS microcarriers that exhibited a textured surface compared with smooth surfaces exhibited by control PLGA microcarriers, PS microcarriers or PS tissue culture plates. Cells cultured on TIPS microcarriers secreted higher amounts of VEGF at all time points up to day 7 compared with cells cultured on tissue culture plastic. It is worth noting that even though the supernatants collected from cellularized TIPS microcarriers were diluted with endothelial growth medium prior to being applied to the in vitro angiogenesis assay, the quantity of VEGF present still exceeded the concentration of the VEGF positive control. The presence of proangiogenic factors in the supernatant collected from AdMSC cultured on TIPS microcarriers accounts for the increase in tubule formation and tubule junctions quantified in the angiogenesis assay. This was supported by the plurality of factors detected by the proteome profiler array. Several of the key proangiogenic factors found to be increased in the pooled supernatants are likely to contribute to the increased angiogenesis observed in vitro. In contrast, the level of multiple proteins in supernatants collected from solid microcarriers were found to be reduced despite being composed of the same polymer, indicating that the textured surface of the TIPS microcarriers rather than its chemical composition contributes to the effect observed. We speculate that the reason for the differences in secretome profile may relate to how AdMSC attached to the different type of PLGA microcarriers. AdMSC formed clusters at the surface of the solid microcarriers (as shown in Figure 2Ciii) rather than a homogenous cell layer over the surface observed with the TIPS microcarriers (Figure 2Civ). Further assessment using planar substrates composed of TIPS and solid polymer are required to investigate the effect of this in more detail. The data from the angiogenesis assay also indicated that conditioned medium obtained from the TC group stimulated a similar response to TIPS polymer 1 and the VEGF positive control, despite the amount of secreted VEGF for the TC control being generally lower than the TIPS microcarriers. The reason for this might relate to other proangiogenic factors in addition to VEGF being secreted into the conditioned medium for the TC group that are not present in the TIPS microcarrier group. TC plastic is optimized to support cell attachment and growth so the secretome in response to attachment to this substrate is likely to differ to the TIPS microcarriers. However, microcarriers composed of TC plastic are not a feasible option for clinical implantation. Therefore, equivalence between implantable PLGA TIPS microcarriers and TC microcarriers still provides a potentially beneficial outcome.

Furthermore, the degradation of the PLGA TIPS microcarriers may contribute to the proangiogenic effect. PLGA degrades into lactate (salt form of lactic acid) and glycolate (salt form of glycolic acid), which in vivo can be metabolized and excreted by the host. Lactic acid has been reported to be an important contributor to angiogenesis in wound healing and to stimulate endothelial cell migration in vitro.^[^ ^48^, ^49^, ^50^ ^]^ For example, subcutaneous implantation of PLGA resulted in sustained local and systemic lactate release stimulated angiogenesis and healing of wounds in ischemic mice.^[^ ^48^ ^]^ Similarly, porous titanium implants coated with PLGA were reported to promote angiogenesis through release of lactic acid as a degradation product.^[^ ^51^ ^]^

Overall, these findings support the notion that the proangiogenic secretome can be directly influenced by the physicochemical properties of the substrate and represents an important parameter to consider when designing implantable microcarriers. The current in vitro proof‐of‐concept study indicates that biodegradable porous microcarriers stimulate the secretion of proangiogenic factors from AdMSC capable of inducing angiogenesis. It is recognized that while the in vitro angiogenesis assay combined with data from the ELISA and proteome angiogenesis array provide a useful indication of this response, the results from this study will need to be validated using suitable in vivo models.

Although higher values of VEGF were measured between days 1–7, the amount of VEGF measured in the supernatants collected at days 9 and 11 were similar between the TIPS polymer groups and the TC group. The reason for this is uncertain but might relate to changes to the physicochemical properties of the microcarriers as they start to degrade under hydrolysis. This is further supported by findings that when the in vitro angiogenesis assay was incubated with supernatant collected from cellularized TIPS microcarriers at a single timepoint, there was an increase in tubule formation compared with tubules formed when incubated with AdMSC secretome collected from different time points. This finding suggests that the temporal profile of the AdMSC secretome influences the angiogenic process. The mechanism underlying the change in composition of the AdMSC secretome might arise from either change to the texture of the TIPS microcarriers as the polymer degrades under hydrolysis during cell culture, which raises the prospect of further refinement and identification of specific surface topographies that induce optimum cell activation. Alternatively, temporal response may result of paracrine signaling pathways arising from the secretome influencing the behavior of the cells. The importance of a temporal control of angiogenesis is recently increasingly been emphasized.^[^ ^52^ ^]^

The stimulatory effect of the material did not appear to affect the phenotype of the AdMSC since cells attached to the surface of the PLGA TIPS microcarriers and subsequently migrated off retained their capacity for trilineage differentiation. This is an important finding since if the substrates led to lineage commitment it would limit future clinical use to implantation of cellularized microcarriers into tissues associated with that lineage.

Given the intended purpose of TIPS microcarriers is to provide an implantable substrate for adhered cells, the cellularized microcarriers were incubated under static conditions for extended periods in vitro to simulate the interplay between cellularized TIPS microcarriers when implanted as a bolus in vivo. Under these conditions AdMSC cultured on TIPS microcarriers readily formed neotissue constructs via cell–cell connections between adjacent microcarriers, demonstrating they are well suited to supporting cell growth. The formation of the tissue construct is likely to have provided a niche for the paracrine interaction of the plethora of factors released by the cells. The importance of cell–cell interactions on paracrine activity was highlighted recently by Qazi et al., where microporous scaffolds that promoted cell–cell interactions enhanced the activity of MSC compared with nano‐porous hydrogels that prevented cell–cell interactions.^[^ ^30^ ^]^ This observation, together with the tunable properties of TIPS microcarriers related to the choice of microcarrier polymer composition, porosity, rate of degradation and size, present further opportunities for tailoring implantable microcarrier substrates that can be designed to enhance the therapeutic effects of MSC mediated by the paracrine factors secreted by these cells, and warrants further investigation.

### 3.2 Future Perspectives

This study provides new insight into the use of material‐based biosystems that can be used to harness and enhance the therapeutic potential of MSC. To verify the proangiogenic potency of the enhanced secretome observed in the current study it will be necessary to investigate the system using suitable in vivo models. This will provide a better understanding of how the secretome influences the complex multicellular processes associated with angiogenesis, as well as the influence of dynamic factors, such as microcarrier degradation and increased vascularization in the local environment, on MSC survival, retention and function. It will also be instructive to conduct longer‐term studies that enable the duration of effect to be observed beyond the time it takes for complete biodegradation of the microcarriers.

Although the AdMSC used in our study are from a well‐characterized commercial source it will be interesting to investigate potential donor‐to‐donor variability and whether MSC derived from other tissues (e.g., umbilical cord, bone marrow, amniotic fluid, and peripheral blood) behave in the same way.

It is generally accepted that the majority of MSC‐mediated therapeutic benefits relate to the secretion of bioactive molecules. Potential therapeutic benefit of cell free MSC‐conditioned supernatants has been suggested for a variety of conditions including cardiovascular disease,^[^ ^53^ ^]^ traumatic brain injury,^[^ ^54^ ^]^ and immunomodulation.^[^ ^55^ ^]^ It is therefore conceivable that an alternative application of the MSC‐TIPS microcarrier system might involve collection of cell‐free conditioned supernatants containing factors and extracellular vesicles from large‐scale bioreactors for cell‐free clinical application.

## 4 Conclusions

The current study demonstrates for the first time that biodegradable hierarchically structured microcarriers suitable for in vivo delivery of AdMSC can be tailored to stimulate the secretion of a proangiogenic secretome. These findings offer a feasible approach for achieving targeted delivery of AdMSC and harnessing their proangiogenic effects to increase neovascularization, which could be used as a new therapy for cardiovascular disease.

## 5 Experimental Section

### Fabrication of Hierarchically Structured PLGA Microcarriers Using Thermally Induced Phase Separation

Hierarchically structured PLGA microcarriers were prepared using thermally induced phase separation (TIPS), as previously described.^[^ ^33^ ^]^ Briefly, PLGA was dissolved in dimethyl carbonate (DMC; Sigma Aldrich) using magnetic stirring overnight. Three different PLGA polymers that differ in the ratio of lactide:glycolide were used (Purasorb PDLG7507 75:25, PDLG5010 50:50, and PDLG8531 85:15; Corbion, Amsterdam, Netherlands). Each polymer was dissolved at different weight:volume (w/v) ratios of DMC: 1% (w/v) PDLG8531 (polymer 1), 5% (w/v) PDLG5010 (polymer 2), and 10% (w/v) PDLG7507 (polymer 3).

Droplets of the polymer solution were generated using a Nisco Encapsulator Unit Var D (Nisco Engineering), fitted with a stainless steel, sapphire‐tipped nozzle (100 µm orifice). The polymer solution was fed into the encapsulator unit through silicone tubing via a syringe pump (Nexus 6000; Chemyx, USA) at a constant rate of 2 mL min^−1^. The vibration frequency of the nozzle was kept at 2.70 kHz and the amplitude of frequency at 100%. The polymer droplets were delivered into a 1 L polypropylene beaker containing liquid nitrogen to achieve thermally induced phase separation of the polymer and solvent before being lyophilized in a freeze drier (Edwards MicroModulyo) for 24 h to achieve sublimation of the frozen solvent. The dried microcarriers were sieved to within a size range of 250–355 µm, which is a similar size range to commercially available microcarriers used for bioprocessing.

### Fabrication of Solid PLGA Microcarriers Using Emulsion Solvent Evaporation Technique

An oil‐in‐water (O/W) emulsion solvent evaporation technique was used for the preparation of solid PLGA microspheres following published protocols.^[^ ^56^, ^57^ ^]^ Accordingly, each polymer was dissolved at different weight:volume (w/v) ratios of DMC: 1% (w/v) PDLG8531 (polymer 1), 5% (w/v) PDLG5010 (polymer 2), and 10% (w/v) PDLG7507 (polymer 3). The PLGA solution was delivered dropwise into an aqueous solution of 0.5% (w/v) polyvinyl alcohol (PVA, average *M*_w_ 30–70 kDa and 87–90% hydrolyzed; Sigma Aldrich) under constant agitation with a rate of 500 rpm achieved by mechanical stirring. The solution was stirred overnight at room temperature followed by collection of the microcarriers after three washes in deionized water. The recovered microcarriers were dried at 37 °C and placed into a vacuum desiccator for storage until further use.

### Characterization of TIPS and Solid PLGA Microcarriers

Samples of the PLGA microcarriers were mounted on aluminum stubs using adhesive carbon tabs and sputter coated with gold (Polaron E5000) and viewed using a Hitachi S3400N SEM at 5 keV. The images acquired were analyzed using an image processing algorithm (ImageJ, National Institutes of Health, Bethesda, MD, USA) to determine the size and shape of the TIPS microcarriers, by evaluating diameter and shape descriptors, including aspect ratio and roundness from top view SEM images.

The number of PLGA microcarriers per unit mass was quantified using a particle image analyzer (Morphologi G3; Malvern Panalytical, UK). Parameters measured included the size and shape of particles using the technique of static image analysis. Due to the high electrostatic surface charge of the TIPS microcarriers, the microcarriers were suspended in cell culture medium prior to analysis. Six replicate measurements were collected per type of microcarrier and the mean number of microcarriers per unit mass was calculated.

### Porosity of TIPS PLGA Microcarriers

The porosity of the TIPS microcarriers was investigated by N_2_ gas adsorption–desorption measurement at 77 K on a Quantachrome Autosorb‐iQC. Prior to measurement, the powder samples (>100 mg) were degassed at 40 °C overnight under dynamic vacuum to ensure minimal adsorbate. The specific surface area and pore size distribution were calculated from the isotherm using the BET and Barrett–Joyner–Halenda (BJH) method, respectively. The total pore volume was estimated from the adsorbing amount of N_2_ at a relative pressure, *P*/*P*_0_, of <0.995.

The surface porosity of the TIPS microcarriers (% total area and pore shape) was determined from top view SEM images.

### Prewetting of TIPS and Solid PLGA Microcarriers Prior to AdMSC Attachment

TIPS PLGA microcarriers were hydrophobic and required prewetting before cell attachment, as previously described.^[^ ^58^ ^]^ The wetting solution consisted of 10% (v/v) absolute ethanol in cell culture medium [Alpha Minimal Essential Medium (aMEM; Gibco) supplemented with 5% (v/v) human platelet lysate (HPL; Stemulate, Cook Regentec, USA) and 2 × 10^−3^ m l‐glutamine (Sigma Aldrich); referred to as proliferation medium]. Dry TIPS and solid PLGA microcarriers were transferred into 7 mL polycarbonate containers and 5 mL of the wetting solution was added. The microcarriers were incubated under rotation in the wetting solution at 37 °C. Successful wetting of the microcarriers was confirmed by their sedimentation in the container. The microcarriers were rinsed twice in fresh culture medium and immediately used for the in vitro experiments.

### Human Adipose‐Derived Mesenchymal Stromal Cells

StemPro human adipose‐derived mesenchymal stromal cells (AdMSC, ThermoFisher Scientific) (passages 2 and 3) were cultured in Alpha Minimal Essential Medium (aMEM; Gibco) supplemented with 5% (v/v) HPL (Cook Regentec).^[^ ^59^ ^]^

### Evaluation of AdMSC Adhesion to TIPS and Solid PLGA Microcarriers

To evaluate cell adhesion to the microcarriers, 0.5 × 10^6^ AdMSC (passages 4 and 5) were mixed with ≈17 500 prewetted PLGA TIPS microcarriers of the three different PLGA compositions (polymers 1, 2, or 3) or solid microcarriers in low‐attachment six‐well cell culture plates (Costar; Corning). Cells were incubated in semidynamic conditions (30 s at 250 rpm h^−1^) on a microplate shaker (SciQuip; UK) for 18 h. After 6 h, unattached cells were removed by rinsing in PBS and the cell–microcarrier combination was fixed in 4% formaldehyde in PBS followed by permeabilization in 0.5% (v/v) Triton X‐100 and staining with phalloidin (Alexa Fluor 647 Phalloidin; ThermoFisher, UK) and Hoechst (1:500; ThermoFisher Scientific) for cytoskeletal and nuclear staining, respectively. Cells were imaged using an inverted fluorescence microscope (Leica DM16000B). Quantification of the number of cells attached to each microcarrier was performed using ImageJ. The number of nuclei per microcarrier was calculated using the “Cell Counter” plugin. Counts were acquired from three separate experiments with ≈60–70 microcarriers counted per experiment. Cell coverage was quantified by calculating the ratio of the cell surface area per microcarrier surface area.

A hanging drop culture plate (Perfecta 3D hanging drop plate; 3D Biomatrix, USA) was used to attach a precise quantity of cells to individual microcarriers. A 40 µL droplet of proliferation medium containing a single microcarrier and 5 × 10^4^ cells mL^−1^ cell was added to individual wells on the plate and incubated for the specified amount of time at 37 °C in 5% CO_2_.

### Long‐Term Cell Culture of AdMSC on TIPS PLGA Microcarriers

To evaluate the effect of extended periods of AdMSC culture on TIPS microcarriers, 0.5 × 10^6^ AdMSC (passages 4 and 5) were seeded onto ≈17 500 microcarriers as described above for each of the three different PLGA TIPS microcarrier compositions. After 18 h, the cellularized TIPS microcarriers were transferred to 24‐well low bind plates and cultured for a further 11 days under static conditions. The confined conditions of the 24‐well plate resulted in the microcarriers forming a multi‐layered 3D structure. The quantity of cells attached the microcarriers at each time point was measured using an automated counter (NC200; Chemometec, Denmark). As a control condition, AdMSC were seeded at 4.5 × 10^4^ cells per well in 2 mL medium on standard PS tissue culture plastic (“TC”) and cultured for further 11 days under static conditions. The use of cell densities greater than this led to rapid confluency and monolayer detachment before the completion of the study. At predetermined timepoints (days 1, 3, 5, 7, 9, and 11) the supernatant from each well was collected for further analysis and replaced by fresh proliferation medium. The quantity of cells was measured in one sample at each time point by DNA quantification using the DNAeasy Blood and Tissue kit (QIAGEN). DNA concentration in the samples was measured at 260 nm wavelength using a Nanodrop 2000c spectrophotometer (Thermo Scientific).

### Viability of AdMSC on TIPS Microcarriers

Viability of AdMSC attached to TIPS microcarriers was assessed using the live–dead cell staining kit (Invitrogen, UK). At day 11, cellularized TIPS microcarriers were incubated with the staining solution for 30 min. Cell staining was observed immediately under a laser scanning confocal microscope (Olympus TIRF). Viable cells were stained with the cell‐permeable live dye (calcein), fluorescing green. Dead cells were stained with both the cell‐permeable live dye and the cell nonpermeable ethidium homodimer‐1, fluorescing red.

The level of cytotoxicity associated with incubating cells attached to the microcarriers was evaluated using the CytoTox 96 nonradioactive cytotoxicity assay (Promega, UK), which measures LDH, a stable intracellular enzyme that is released into the culture supernatant following cell lysis. The assay was performed according to manufacturer’s instructions. Three biological replicates were used per timepoint and per condition.

### VEGF Secretion from AdMSC on TIPS Microcarriers

The quantity of human VEGF in the cell culture supernatants collected from cellularized TIPS microcarriers at different timepoints was measured by ELISA (DuoSet, R&D Systems, Minneapolis, MN). Supernatant from cells cultured on tissue culture plates or TIPS microcarriers without cells were included as control samples. Three biological replicates were collected from each timepoint and condition.

### In Vitro Angiogenesis Assay of Supernatants Collected from AdMSC on TIPS Microcarriers

The angiogenic activity of supernatants collected from cellularized TIPS microcarriers was determined using V2a Kit‐Vasculogenesis to Angiogenesis (Cellworks, Buckingham, UK). The supernatants from cellularized microcarriers or control samples (cells cultured on tissue culture plastic) at different points were diluted 1:1 with V2a growth medium before addition to the culture (*n* = 3 wells per condition). The culture medium in each well was replaced every two days with either conditioned medium collected at 2 day intervals or with conditioned medium collected at day 3 only.

Positive (VEGF 2 ng mL^−1^) and negative (Suramin 20 × 10^−6^ m) control samples were included as recommended by the assay manufacturer (*n* = 2 wells per condition). At day 14, the cells were fixed in 70% ice‐cold ethanol and stained with mouse antihuman CD31 primary antibody and goat antimouse IgG‐alkaline phosphatase conjugated secondary antibody, according to manufacturer instructions. Images of the stained tubules were acquired 4× magnification using a stereomicroscope (Leica MZ10F; *n* = 4 fields of view per well). Tubule formation was analyzed using Cellworks Image Analysis Software, AngioSys 2.0 (Cellworks).

### Screening the Angiogenic Secretome from AdMSC on TIPS Microcarriers

Supernatants were collected from wells in the hanging drop plates containing cellularized PLGA TIPS microcarriers composed of polymer 3, solid PLGA microcarriers composed of polymer 3, or PS microcarriers (PS300K, Phosphorex, USA) at days 1, 4, 7, and 10 and replenished with fresh culture medium. The collected supernatants were immediately frozen until further analysis. The relative amount of soluble angiogenesis‐related proteins in the pooled supernatants was measured using the Human Proteome Profiler Angiogenesis Array (ARY007, R&D Systems, UK), following manufacturer’s instructions. The optical density from chemiluminescent signal of each analyte dot was calculated in ImageJ and the values plotted using GraphPad Prism (GraphPad Software, San Diego, USA).

### AdMSC Migration and in Vitro Trilineage Differentiation

After 11 days incubation in low attachment 24‐well plates, cellularized TIPS microcarriers were transferred into 6‐well tissue cultures plates and incubated for 4–6 days to allow cells to migrate from the microcarriers onto the plates and reach 60–80% confluency. The migrated cells were detached using trypsin‐EDTA and seeded into 12‐well tissue culture plates at densities suitable for trilineage differentiation.

For adipogenic differentiation, AdMSC were seeded at 1 × 10^4^ cells cm^−2^ and cultured in MesenPRO RS Medium. After 3 days, the culture medium was replaced with StemPro Adipogenesis Differentiation medium (ThermoFisher Scientific) for 12–14 days, with the medium replenished every 3–4 days. Adipogenic differentiation and the presence of lipids in the cells was evaluated by staining with LipidTOX (HCS LipidTOX Green Neutral Lipid Stain, ThermoFisher Scientific).

For osteogenic differentiation, AdMSC were seeded at 5 × 10^3^ cells cm^−2^ and cultured in MesenPRO RS Medium. After 3 days, culture medium was replaced with StemPro Osteogenesis Differentiation medium (ThermoFisher Scientific) for 21 days, with the medium replenished every 3–4 days. Osteogenic differentiation was evaluated by staining with Alizarin Red S (ACROS Organics). Quantitative evaluation of the stain has been performed by dye extraction using an aqueous solution of 20% (v/v) methanol (Acros Organics, USA) and 10% (v/v) acetic acid (Acros Organics, USA). The absorbance was measured at 450 nm wavelength.

For chondrogenic differentiation, micromass culture was performed by seeding 5 µL droplets of a high concentration cell suspension (1.6 × 10^7^ cells mL^−1^) in the center of a well of a 48‐well plate. After 2 h incubation at 37 °C in 5% CO_2_, chondrogenesis medium (ChondroMAX Differentiation Medium; Sigma Aldrich) was added to the well for 14 days, with the medium replenished every 2–3 days. Chondrogenic differentiation was evaluated by staining with Alcian Blue dye (Sigma Aldrich).

### Ultrastructural Characterization of AdMSC Attachment to TIPS Microcarriers

The interaction of cells growing on the surface of TIPS microcarriers was analyzed by SEM. After 24 h culture, cellularized TIPS microcarriers were washed with PBS and fixed with 4% formaldehyde in PBS for 2 h at 4 °C. The samples were washed with PBS and dehydrated in serially graded ethanol solutions (20–100% v/v) for 5 min each. The samples were immersed in hexamethyldisilazane (Sigma Aldrich) for 2 min and were left to dry at room temperature in a desiccator. Samples were sputter coated with gold (Polaron E5000) and imaged using a Hitachi S3400N SEM at 5 keV.

### Statistical Analysis

Data were subjected to one‐way or two‐way ANOVA followed by Tukey’s tests for multiple comparisons between pairs of means, using commercially available software (GraphPad Prism). Statistically significant difference between experimental results was indicated by *p* < 0.05 (*), *p* < 0.01 (**), *p* < 0.001 (***), and *p* < 0.0001 (****). Results are expressed as mean ± standard error of the mean (SEM; *n* = 3) or as mean ± STDEV.

## Conflict of Interest

The authors declare no conflict of interest.

## Supporting information

Supporting Information
