# Supplementary material for: Promotion of Proangiogenic Secretome from Mesenchymal Stromal Cells via Hierarchically Structured Biodegradable Microcarriers
Source: Adv Biosyst. 2020 Jun 8;4(7):2000062. doi: 10.1002/adbi.202000062 (PMC8425330; doi:10.1002/adbi.202000062)
Supplement: Supplementary file 1 — Supporting Information [file ADBI-4-2000062-s001.pdf]

Copyright WILEY-VCH Verlag GmbH & Co. KGaA, 69469 Weinheim, Germany, 2020.

ADVANCED BIOSYSTEMS

Supporting Information

for *Adv. Biosys.*, DOI: 10.1002/adbi.202000062

Promotion of Proangiogenic Secretome from Mesenchymal Stromal Cells via Hierarchically Structured Biodegradable Microcarriers

*Chara Simitzi, Eseelle Hendow, Zhuangnan Li, and Richard M. Day**

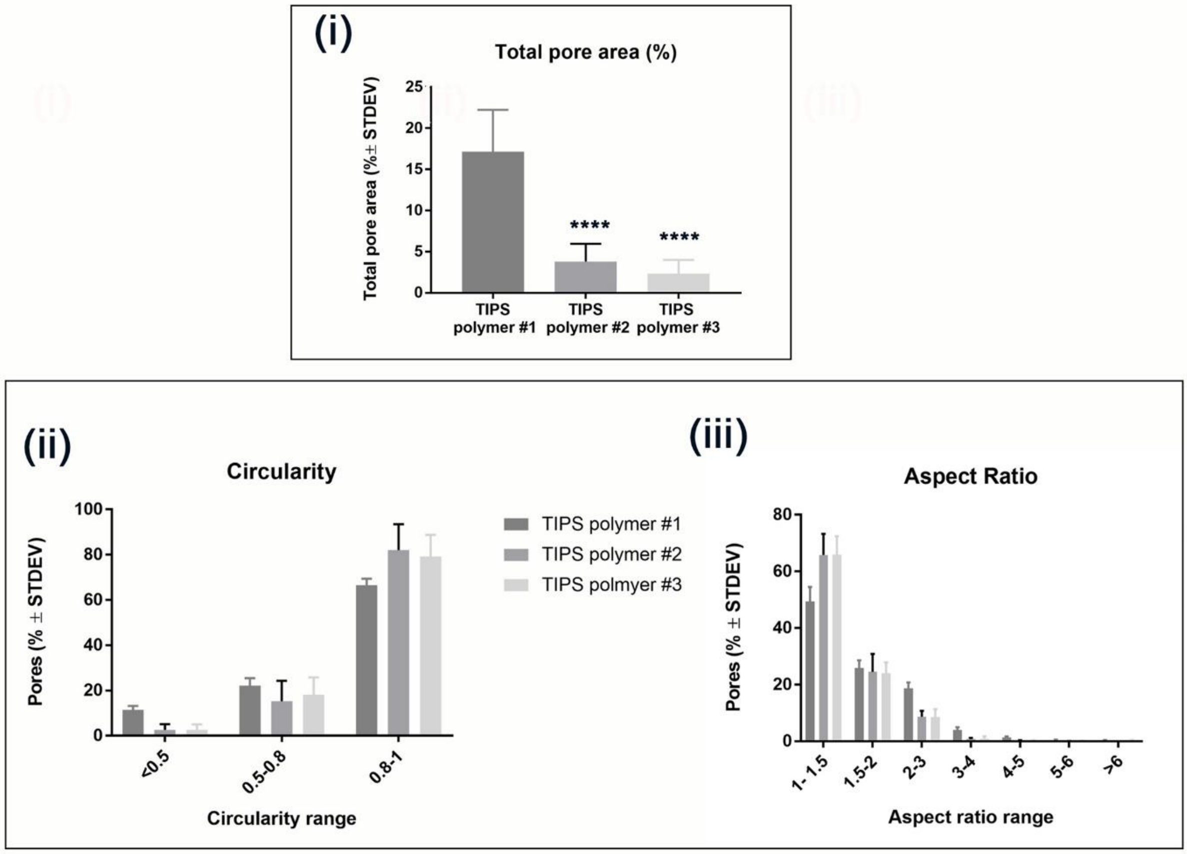

Supp. Figure 1: Quantitative evaluation of morphological characteristics of the surface porosity of TIPS PLGA microcarriers, total area (i), circularity (ii) and aspect ratio (iii); results are expressed as mean % ± standard deviation, STDEV (approximately n=15 TIPS microcarriers per polymer type); the significance level (p) was calculated using the One way ANOVA followed by Tukey test for multiple comparisons between the TIPS microcarriers of polymer #2 and #3 with these of polymer #1 (**** $p<0.0001$).

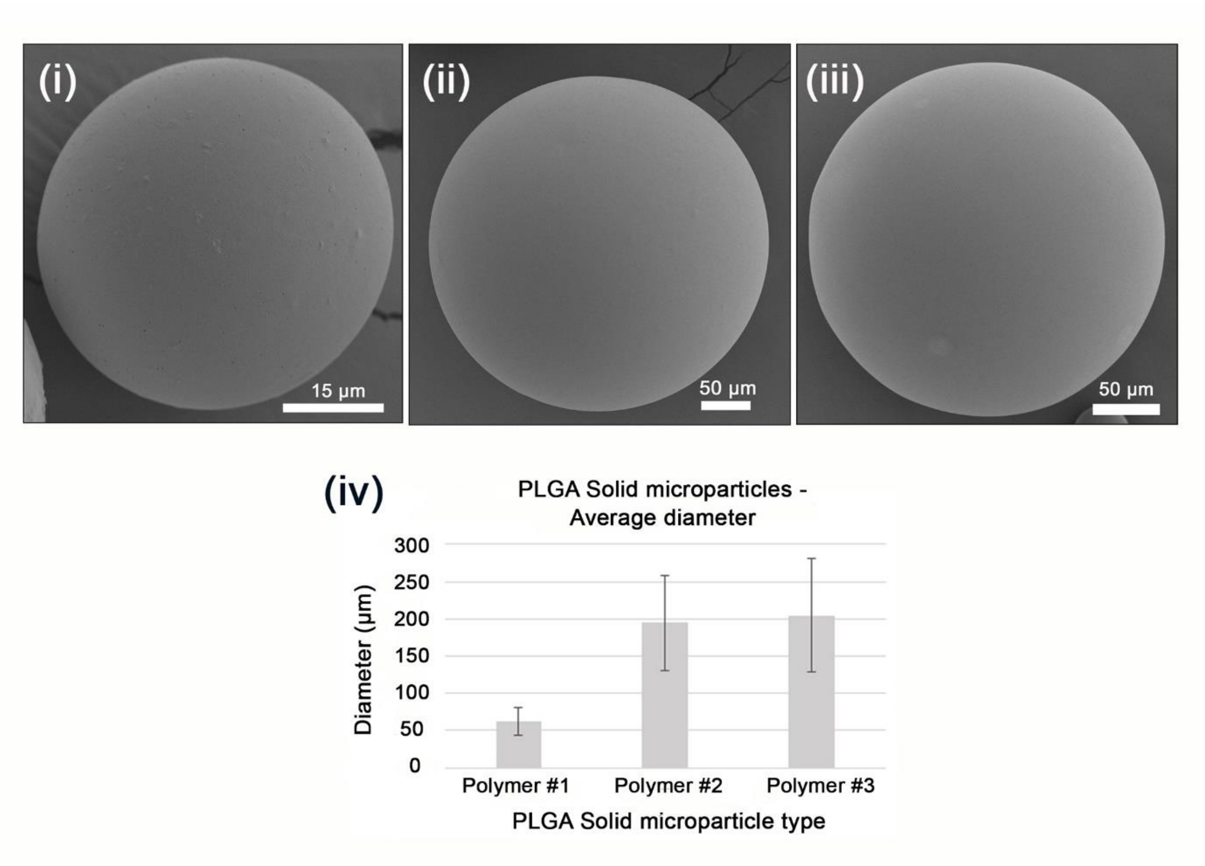

Supp. Figure 2: Solid PLGA microcarriers with smooth surface fabricated by emulsion solvent evaporation method. SEM images of microcarriers of polymer #1 (i), #2 (ii) and #3 (iii); quantitative evaluation of the mean diameter of the solid microcarriers (iv).

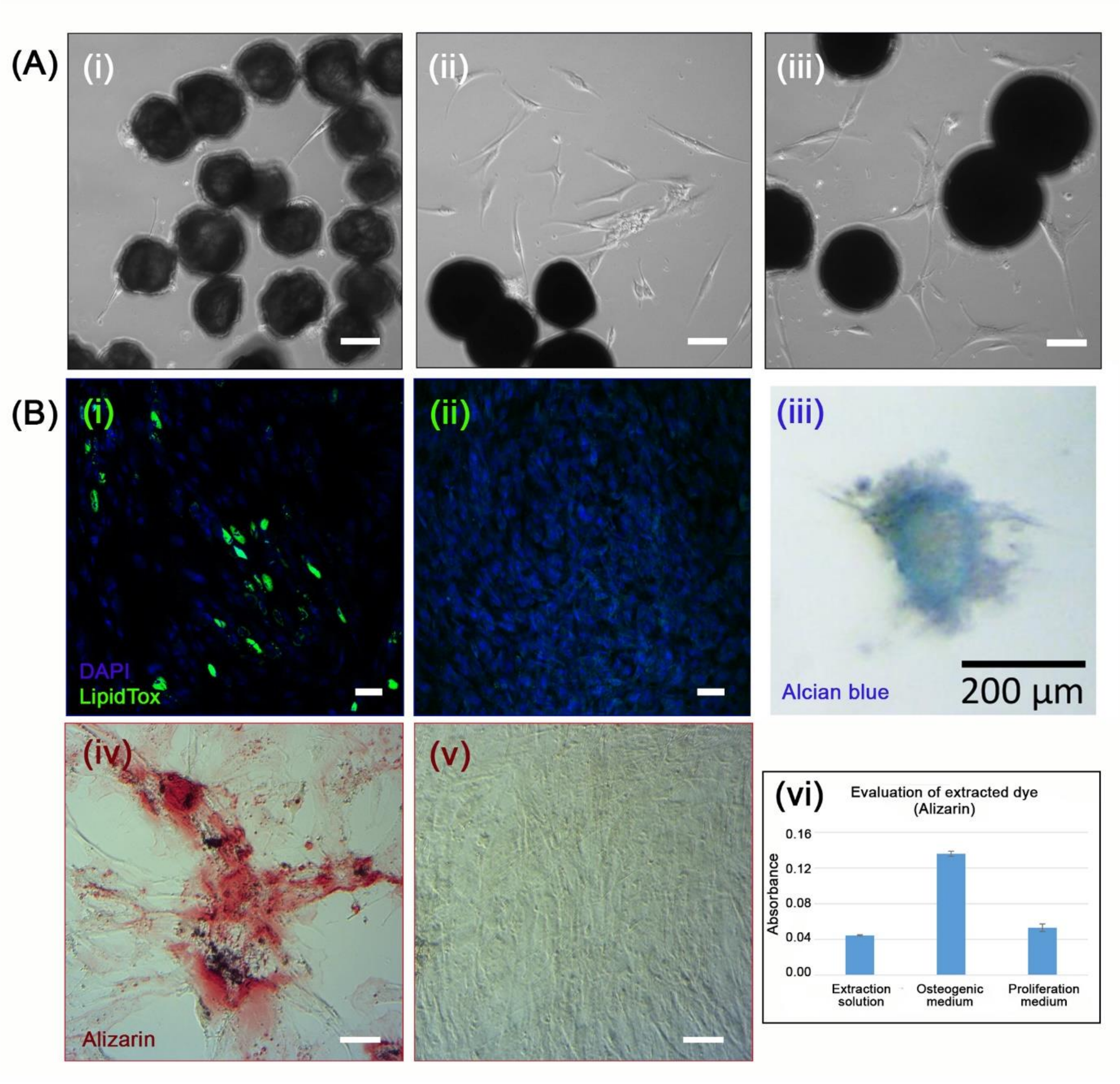

Supp. Figure 3: Cell migration and differentiation study. (A) Light microscopy images of AdMSC migrating off TIPS microcarriers composed of polymer #1 (i), polymer #2 (ii) and polymer #3 (iii) microcarriers after 11 days culture on the microcarriers (scale bar: 100 µm). (B) Differentiation study of MSC migrated from the TIPS microcarriers onto tissue culture plastic. AdMSC incubated in adipogenic (i) and proliferation (ii) medium. Lipid droplets were stained with LipidTOX (green) and cell nuclei with Hoechst (blue) (scale bar: 50 µm); (iii) a cell spheroid of AdMSC cultured for 14 days in chondrogenic differentiation medium and stained with Alcian blue (blue); (iv) AdMSC incubated in osteogenic and (v) proliferation medium stained with Alizarin (red) (scale bar: 100 µm); quantification of alizarin dye extracted from AdMSC at day 21(vi).
